# The association between body roundness index and asthma risk in middle-aged and elderly people: a retrospective cohort study based on CHARLS database

**DOI:** 10.1186/s12890-026-04233-y

**Published:** 2026-03-10

**Authors:** Liping Huang, Jinming Zhang, Qingmei Wang, Haohua Huang, Yuhan Du, Qi Yu, Dongyu Liu, Jie Chen, Xiaoxiao Jiang, Yanqun Li, Jinzhong Zhuo, Baosong Xie, Shaoxi Cai, Hangming Dong

**Affiliations:** 1 Shengli Clinical Medical college of Fujian Medical University, Fuzhou, China; 2 Department of Respiratory and Critical Care Medicine, Fuzhou University Affiliated Provincial Hospital, Fuzhou, China; 3 Department of Respiratory and Critical Care Medicine, Nanfang Hospital, Southern Medical University, Guangzhou, China; 4 Department of Respiratory and Critical Care Medicine, Ganzhou People’s Hospital, Ganzhou, China

**Keywords:** Body Roundness Index, Asthma, Visceral Adiposity, Cohort study, CHARLS

## Abstract

**Background:**

The prevalence of asthma is rising globally, posing a significant public health burden. While obesity is a well-established risk factor for asthma, traditional metrics like Body Mass Index (BMI) have limitations in distinguishing visceral adiposity. The Body Roundness Index (BRI) is a novel geometric indicator of central obesity, but its longitudinal association with asthma risk in middle-aged and elderly populations remains unclear.

**Methods:**

We conducted a retrospective cohort study using data from the China Health and Retirement Longitudinal Study (CHARLS) covering the period from 2011 to 2020. A total of 7,754 participants aged 45 years and older, free of respiratory diseases at baseline, were included. Multivariable Cox proportional hazards models and restricted cubic spline (RCS) analyses were employed to evaluate the association between BRI and the incidence of asthma.

**Results:**

During a 7-year follow-up, 521 incident asthma cases were identified. In fully adjusted models, elevated BRI was significantly associated with an increased risk of asthma (Hazard Ratio [HR] = 1.39, 95% CI: 1.388–1.393 per SD increase). RCS analysis revealed a non-linear U-shaped relationship with a distinct inflection point at a BRI of 4.12. Beyond this threshold, the risk of asthma increased substantially (HR = 1.75, 95% CI: 1.34–2.27). Subgroup analyses demonstrated that the association was more pronounced in females and urban residents.

**Conclusion:**

BRI is significantly associated with the risk of asthma in middle-aged and elderly Chinese adults, exhibiting a non-linear dose-response relationship. As a superior marker of visceral adiposity, BRI may serve as a simple and effective tool for identifying high-risk individuals, particularly women, to facilitate early screening and prevention strategies for asthma.

## Background

The global prevalence of asthma has been increasing in recent years, with approximately 300 million individuals affected worldwide, as reported in the Global Asthma Report [1–3]. This rising prevalence shows significant variation among older adults [4]. In China, approximately 4.2% of adults aged 20 and older are diagnosed with asthma [5], and this prevalence is expected to rise due to changing lifestyles. Adults with asthma have a significantly higher all-cause mortality rate compared to non-asthmatics [6]. Besides impacting patients’ quality of life, asthma also imposes a substantial burden on healthcare resources [4, 7], underscoring the importance of addressing its associated risk factors.

The Body Roundness Index (BRI) is an innovative indicator of obesity distribution, calculated using waist circumference and height, which offers a more comprehensive evaluation of an individual’s fat distribution [8]. Research indicates that BRI may provide a superior assessment of the health impacts associated with fat compared to the traditional Body Mass Index (BMI) [8]. Elevated BRI has been linked to a higher incidence of various diseases, including cardiovascular disease and diabetes [9, 10]. Moreover, BRI has shown efficacy in predicting metabolic syndrome and cardio-metabolic risk factors across diverse populations, although its predictive accuracy may differ by demographic and age group [11–13].

In recent years, obesity has been identified as a significant risk factor for asthma, particularly in adults [14, 15]. The prevalence of asthma is notably higher among obese individuals [16, 17]. Conventional anthropometric measures, such as BMI, often inadequately assess the fat distribution, which may limit their effectiveness in predicting obesity-related health risks [18]. Data indicate that the physical location of fat significantly influences disease risk. Higher concentrations of visceral adipose compared to subcutaneous adipose tissue are associated with increased metabolic risks [19]. The BRI has gained attention as an innovative assessment tool that offers a more comprehensive evaluation of body shape and fat distribution, especially visceral fat [20]. Elevated BRI has been associated with declines in respiratory function [21], likely due to excessive fat accumulation and systemic inflammation [22, 23]. Despite substantial evidence indicating a link between obesity and asthma, direct studies assessing BRI as a specific measure in relation to asthma are limited. Therefore, this study aims to employ a retrospective cohort design to investigate the association between BRI and adult asthma using the China Health and Retirement Longitudinal Study (CHARLS) database.

## Method

### Data source and participants

**Fig. 1:**
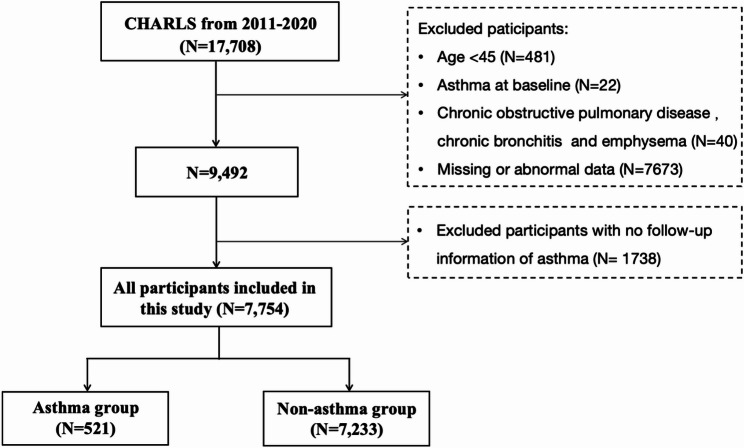
Flowchart for screening eligible participants in the study from CHARLS 2011–2020. Note: Exclusion due to “abnormal data” refers to biologically implausible anthropometric measurements (waist circumference < 50 cm or > 150 cm; height < 120 cm or > 210 cm). CHARLS, China Health and Retirement Longitudinal Study

This study utilized data from the CHARLS database [24], a nationally representative longitudinal survey focusing on the health, retirement, and economic conditions of individuals aged 45 and older in mainland China. A total of 17,708 respondents participated in the CHARLS surveys that were carried out between 2011 and 2020. Our study included 7,754 participants after applying the following exclusion criteria: (1) age < 45 years; (2) self-reported physician-diagnosed asthma at baseline; (3) confirmed chronic pulmonary diseases (including chronic obstructive pulmonary disease, chronic bronchitis, or emphysema) based on medical records; (4) missing data on key variables (waist circumference, height, or asthma status), or “abnormal data” (biologically implausible values defined as waist circumference < 50 cm or > 150 cm, or height < 120 cm or > 210 cm-thresholds consistent with standard anthropometric quality control protocols in large-scale epidemiological studies) [24]; and (5) absence of follow-up data for asthma incidence. For participants with missing data on other covariates, multiple imputation by chained equations (MICE) was performed to address missingness and minimize selection bias [25]. The detailed participant selection procedure is illustrated in Fig. 1. Asthma cases were identified using a validated composite definition requiring both (1) self-reported physician diagnosis of asthma and (2) confirmation via medical records with corresponding International Classification of Diseases (ICD) codes. To further minimize misclassification (false positives), we rigorously excluded participants with baseline chronic pulmonary diseases (e.g., COPD, chronic bronchitis, emphysema) as confirmed by medical records.

### Variables

The primary exposure variable in this study was BRI, calculated using height and waist circumference (WC) data from the CHARLS baseline survey (2011 wave). BRI, a recently developed index of abdominal obesity, was recorded as a continuous variable. The BRI was calculated as follows [8]:364.2–365.5 × √(1 - ((Waist Circumference/(2π))²)/(0.5 × Height)²). For Cox regression modeling, the continuous BRI variable was z-score standardized [(BRI − cohort mean)/SD] to enhance model convergence and interpretability of hazard ratios. However, to facilitate clinical interpretation, thresholds and inflection points are presented as raw BRI values derived from reverse transformation of the Z-scores.The outcome variable was the incidence of asthma, determined by self-reports and confirmed through medical records, using ICD criteria. Covariates were selected using a dual-criteria strategy integrating clinical relevance and statistical model performance. Initially, candidate variables were pre-specified based on established biological plausibility and prior evidence linking them to asthma risk or BRI, including age, gender, BMI, living place, educational level, marital status, insurance status, hypertension, diabetes, dyslipidemia, smoking status, and physical activity. Additionally, we performed a sensitivity analysis by adjusting for depressive symptoms measured with the CES-D 10 scale available in CHARLS, to examine potential confounding by mental health status. Subsequently, a stepwise selection procedure guided by the Akaike Information Criterion (AIC) was applied to optimize the final covariate set, balancing model fit and parsimony to mitigate overfitting risk. To address potential selection bias associated with missing covariate data and maximize statistical power, missing values for covariates were imputed using the multiple imputation method based on the assumption of missing at random (MAR).

### Statistical analysis

In this study, continuous variables were expressed as mean ± standard deviation (for normal distributions) or median (interquartile range); categorical variables were expressed as frequency and percentage. We used the chi-square test (for categorical variables), Student’s t-test (for normal distributions), or Mann-Whitney U test (for skewed distributions) to compare differences among groups (see Table 1). The association between the BRI and asthma incidence was analyzed through the following steps:

**Table 1:** Baseline characteristics of participants

Characteristic	Low BRI (*N* = 3164)	Middle BRI (*N* = 3157)	High BRI (*N* = 3171)	*P*-value
Age, years, means ± SD	59.59 ± 9.17	59.31 ± 9.15	60.69 ± 9.35	< 0.001
Gender, n (%)				< 0.001
Male	2035(64.34%)	1500(47.56%)	888 (28.03%)	
Female	1128(35.66%)	1654(52.44%)	2280(71.97%)	
BMI, kg/m², means ± SD	20.59 ± 2.71	23.20 ± 2.70	26.74 ± 3.48	< 0.001
BMI group, kg/m², n (%)				< 0.001
≤ 28	3111(98.82%)	3089(98.13%)	2163(68.47%)	
> 28	37 (1.18%)	59 (1.87%)	996 (31.53%)	
Living Place, n (%)				< 0.001
Rural	2910(92.03%)	2822(89.47%)	2759(87.12%)	
Urban	252 (7.97%)	332 (10.53%)	408 (12.88%)	
Educational level, n (%)				< 0.001
Elementary school and below	2206(69.74%)	2172(68.82%)	2353(74.23%)	
Middle/high/vocational school	913 (28.87%)	939 (29.75%)	786 (24.79%)	
College and above	44 (1.39%)	45 (1.43%)	31 (0.98%)	
Marital Status, n (%)				0.212
Single (widowed, divorced, never)	513(16.21%)	523 (16.57%)	564 (17.79%)	
Married	2651(83.79%)	2634(83.43%)	2606(82.21%)	
Insurance, n (%)				< 0.001
No insurance	171 (5.41%)	173 (5.48%)	191 (6.03%)	
New Cooperative Medical Scheme	2630(83.20%)	2453(77.75%)	2437(76.90%)	
Rural Cooperative Medical Scheme	27 (0.85%)	47 (1.49%)	44 (1.39%)	
Other	333(10.53%)	482 (15.28%)	497 (15.68%)	
Hypertension, n (%)				0.016
No	3146(99.43%)	3134(99.27%)	3133(98.80%)	
Yes	18 (0.57%)	23 (0.73%)	38 (1.20%)	
Diabetes, n (%)				0.003
No	3163(99.97%)	3150(99.78%)	3157(99.56%)	
Yes	1 (0.03%)	7 (0.22%)	14 (0.44%)	
Dyslipidemia, n (%)				0.102
No	3161 (99.91%)	3150(99.78%)	3160(99.65%)	
Yes	3 (0.09%)	7 (0.22%)	11 (0.35%)	
Smoking Status, n (%)				< 0.001
Non-smoker	1445 (45.74%)	1921(60.89%)	2352(74.22%)	
Current smoker	1622 (51.35%)	1120(35.50%)	715 (22.56%)	
Quit smoking	92 (2.91%)	114 (3.61%)	102 (3.22%)	
Physical Activity, n (%)				0.925
Inactive	141 (4.46%)	147 (4.66%)	139 (4.38%)	
Minimally Active	405 (12.80%)	394 (12.48%)	424 (13.37%)	
Moderately Active	403 (12.74%)	426 (13.49%)	403 (12.71%)	
Vigorously Active	296 (9.36%)	273 (8.65%)	287 (9.05%)	
Not record	1919 (60.65%)	1917(60.72%)	1918(60.49%)	0.686
Depression, n(%)				
No	1747(62.93%)	1721(61.82%)	1765(62.52%)	
Yes	1029(37.07%)	1063(38.18%)	1058(37.48%)	

*Abbreviation*: *BRI* Body roundness index, *BMI* Body mass index

*P*-values indicate the statistical significance of differences among groups

- Step 1: Univariate and multivariate Cox proportional hazards regression models were applied to assess the association between BRI and incident asthma, accounting for the time-to-event nature of the outcome. Three models were constructed: Model 1 with no covariate adjustment; Model 2 adjusted for sociodemographic data (age, gender, educational level, living place, marital status, insurance); and Model 3 further adjusted for BMI, hypertension, diabetes, dyslipidemia, smoking status, physical activity and depression (Table 2).These models aimed to explore the changes in BRI effect values under different adjustment strategies to assess the robustness of the results.
- Step 2: Assessment of Nonlinearity and Threshold Effect.

**Table 2:** Weighted cox proportional hazards regression analysis of the association between BRI and asthma risk

Models	Crude model		Model 1		Model 2	
	HR (95% CI)	*P*-value	HR (95% CI)	*P*-value	HR (95% CI)	*P*-value
BRI	1.079 (1.078, 1.081)	< 0.00001	1.131 (1.129, 1.132)	< 0.00001	1.390 (1.388, 1.393)	< 0.00001
BRI Quartile						
Q1	Reference		Reference		Reference	
Q2	0.732 (0.731, 0.733)	< 0.00001	0.786 (0.785, 0.787)	< 0.00001	0.810 (0.809, 0.811)	< 0.00001
Q3	0.762 (0.761, 0.763)	< 0.00001	0.891 (0.890, 0.892)	< 0.00001	0.981 (0.980, 0.983)	< 0.00001
Q4	1.075 (1.074, 1.077)	< 0.00001	1.179 (1.177, 1.180)	< 0.00001	1.455 (1.452, 1.458)	< 0.00001
P for trend	1.034 (1.033, 1.034)	< 0.00001	1.065 (1.065, 1.066)	< 0.00001	1.126 (1.125, 1.127)	< 0.00001

Crude model: not adjusted; Model 1: adjusting for age, gender, educational level, living place, marital status, insurance; Model 2: adjusting for Model 1 + BMI, hypertension, diabetes, dyslipidemia, smoking status, physical activity, depression. *HR* Hazard Ratio, *CI* Confidence Interval, *BRI* Body roundness index

To address the potential non-linear relationship between BRI and asthma incidence, we employed a two-stage modeling strategy. First, a preliminary linear Cox proportional hazards model (termed Model I) was fitted to assess the plausibility of a linear association. However, given that the linearity assumption was rejected by the log-likelihood ratio test (*P* < 0.001), we proceeded to characterize the non-linear dose-response relationship using generalized additive models (GAMs). Smooth curve fitting was performed using penalized cubic splines with 3 degrees of freedom (equivalent to 2 internal knots at default quantiles) to balance flexibility with the risk of overfitting. Subsequently, to determine the optimal threshold for the non-linear effect, we utilized a binary recursive partitioning algorithm. This method identified the inflection point (K) that maximized the log-likelihood ratio statistic between model segments. Based on the point yielding the most significant improvement in model fit (*P* < 0.001 for likelihood ratio test), the final inflection point was determined to be 4.12. Consequently, a two-piecewise linear Cox regression model (Model II) was constructed as the primary analysis to calculate the effect sizes on both sides of the inflection point. For sensitivity analysis, the standard linear model was formally compared with the piecewise model using the likelihood ratio test to confirm which model provided a superior explanation of the association between BRI and asthma incidence. To ensure the robustness of data analysis, a sensitivity analysis was conducted by converting BRI into a categorical variable and calculating the *P* for trend. This aimed to validate the results of BRI as a continuous variable and evaluate the potential for nonlinearity.

In this study, we applied the “BIO_WEIGHT2” sampling weight provided by the CHARLS team, which is specifically constructed for analyses involving the biomarker subsample. “BIO_WEIGHT2” integrates the original household-level sampling probabilities, adjusts for individual non-response during biomarker data collection, and further calibrates to national population benchmarks for age, sex, and urban/rural residence to ensure representativeness of the Chinese population aged 45 and older [24]. To account for the complex survey design and produce unbiased hazard ratio estimates, all Cox proportional hazards models were fitted using the “svycoxph” function from the R “survey” package (version 4.2), which implements weighted partial likelihood estimation under stratified cluster sampling. This approach corrects for unequal selection probabilities and non-response bias, thereby enhancing the generalizability of our findings.

To investigate potential heterogeneity in the association between BRI and asthma risk, stratified analyses were performed based on age ( ≦ 65 vs. >65 years), gender (Male vs. Female), living place (Rural vs. Urban), and other baseline characteristics (BMI, education, insurance, hypertension, diabetes, and smoking status). Interaction terms were formally introduced into the Cox models to test for the statistical significance of effect modification across subgroups by calculating the *P*-value for interaction. All other analyses were conducted using the R statistical software package (http://www.R-project.org, The R Foundation). P values less than 0.05 (two-sided) were considered statistically significant.

## Results

### Baseline characteristics of the participants

Our analysis identified significant associations between BRI and various demographic and health-related variables. Specifically, mean age increased with BRI, ranging from 59.59 years in the low BRI group to 60.69 years in the high BRI group (*P* < 0.001). Gender distribution varied markedly, with males comprising 64.34% of the low BRI group, decreasing to 28.03% in the high BRI group (*P* < 0.001), indicating a corresponding rise in female representation. Additionally, the high BRI group exhibited a higher BMI, with values averaging 26.74 compared to 20.59 in the low BRI group (*P* < 0.001). Rural residency was more prevalent among individuals with lower BRI, and lower education levels were correlated with higher BRI (*P* < 0.001). Furthermore, non-smokers constituted 74.6% of the high BRI group (*P* < 0.001). Although significant differences were detected for hypertension (*P* = 0.012) and diabetes (*P* = 0.003) across BRI categories, the prevalence of asthma displayed an increasing trend with BRI levels: 6.6% in the low BRI group, 5.9% in the medium group, and 7.6% in the high group. This trend, while not statistically significant (*P* = 0.053), suggests a potential association between BRI and asthma that warrants further investigation(As shown in Table 1). 

### Association between BRI and asthma risk

**Fig. 2:**
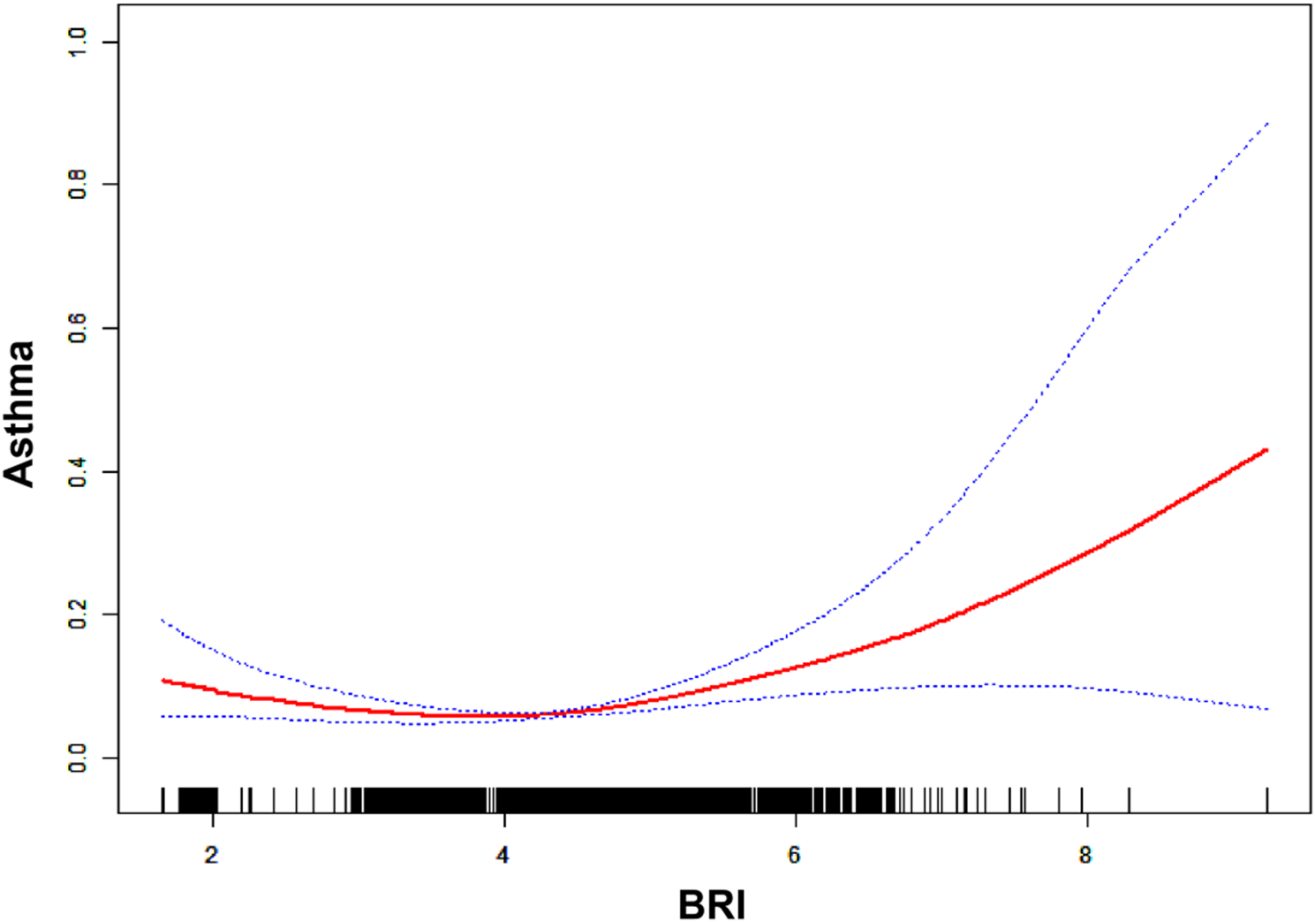
Smooth curve fitting for probing the relationship between BRI and asthma

Consistent with our analytical plan for time-to-event data, we examined the relationship between BRI and asthma risk using Cox proportional hazards regression models. Results revealed a significant association between increased BRI and elevated asthma risk. In the non-adjusted model, each unit increase in BRI was associated with a 7.9% increase in asthma risk [Hazard Ratio(HR): 1.079, 95% Confidence Interval(CI): 1.078, 1.081, *P* < 0.00001]. The Adjusted Model I (adjusting for age, sex, education, marital status, insurance, and living place) showed a 13.1% increased risk (HR: 1.131, 95% CI: 1.129, 1.132, *P* < 0.00001). Adjusted Model II further accounted for BMI, hypertension, diabetes, dyslipidemia, smoking status, physical activity and depression, demonstrating a 39% increase in risk per unit BRI (HR: 1.390, 95% CI: 1.388, 1.393, *P* < 0.00001), highlighting the potential importance of BRI in the pathogenesis of asthma. In the quartile analysis, using the first quartile (Q1) as the reference, the second (Q2) and third (Q3) quartiles exhibited hazard ratios below 1, while the fourth quartile (Q4) showed an elevated risk (HR: 1.455, 95% CI: 1.452–1.458). Rather than implying a simple protective effect of intermediate BRI, this pattern—characterized by lower risk in intermediate groups followed by a sharp increase in the highest quartile—suggests a non-linear, U-shaped association between BRI and asthma prevalence. This statistical non-linearity was subsequently confirmed by our generalized additive model and restricted cubic spline analyses (Fig. 2), which visually demonstrated the complex dose-response relationship. This trend was corroborated by the continuous BRI analysis, which consistently showed a positive correlation between BRI increments and asthma risk (As shown in Table 2).

### Nonlinear relationship between BRI and asthma risk

The scatter plot in Fig. 2 highlights the observed data points (indicated by open circles) alongside smoothed fitted curves (represented by solid lines), which illustrate the trend of increasing asthma prevalence as related to changes in BRI. The shaded regions around the curves denote the 95% CI, providing a measure of reliability for the observed trends. The findings demonstrate a pronounced non-linear rise in asthma prevalence with higher BRI values, indicating that BRI could serve as a valuable biomarker for assessing the risk of asthma.

As a preliminary assessment of linearity, we first fitted a standard linear Cox model (Model I) across the entire BRI range. This model yielded a non-significant hazard ratio of 1.15 (95% CI: 0.94, 1.41; *P* = 0.1738), suggesting that a simple linear relationship was inadequate. Given the strong evidence of nonlinearity from our generalized additive model (*P* < 0.001 for likelihood ratio test comparing linear vs. nonlinear fit), we then applied a two-piecewise linear Cox regression model (Model II) with a data-driven threshold at K = 4.12. Below this threshold (BRI < 4.12), the effect size was 0.71 (95% CI: 0.54, 0.92; *P* = 0.0102), indicating a statistically significant inverse relationship between BRI and asthma risk. Conversely, above the breakpoint (BRI > 4.12), the effect size markedly increased to 1.75 (95% CI: 1.34, 2.27; *P* < 0.0001), demonstrating a strong positive association between BRI and asthma incidence. Additionally, the log-likelihood ratio test yielded a *P*-value of < 0.001(As shown in Table 3), affirming the enhanced fit of the model that accounts for the breakpoint, thereby underscoring the robustness of the associations ascertained.

**Table 3:** Nonlinear relationship between BRI and asthma risk

Model	Effect Description	HR (95% CI)	*P*-value
Model I	Linear Effect	1.15 (0.94, 1.41)	0.1738
Model II	Threshold (K = 4.12)		
< K	Effect 1	0.71 (0.54, 0.92)	0.0102
> K	Effect 2	1.75 (1.34, 2.27)	< 0.0001
Likelihood Ratio Test			< 0.001

Outcome variable: asthma; exposure variable: Body Roundness Index (BRI); Model I represents the initial linear assumption test; Model II is the final piecewise model selected based on significant nonlinearity (likelihood ratio test *P* < 0.001). Covariates adjusted: age, gender, educational level, living place, marital status, insurance, BMI, hypertension, diabetes, dyslipidemia, smoking status, physical activity, depression. *HR* Hazard Ratio, *CI* Confidence Interval

### Subgroup analysis

**Fig. 3:**
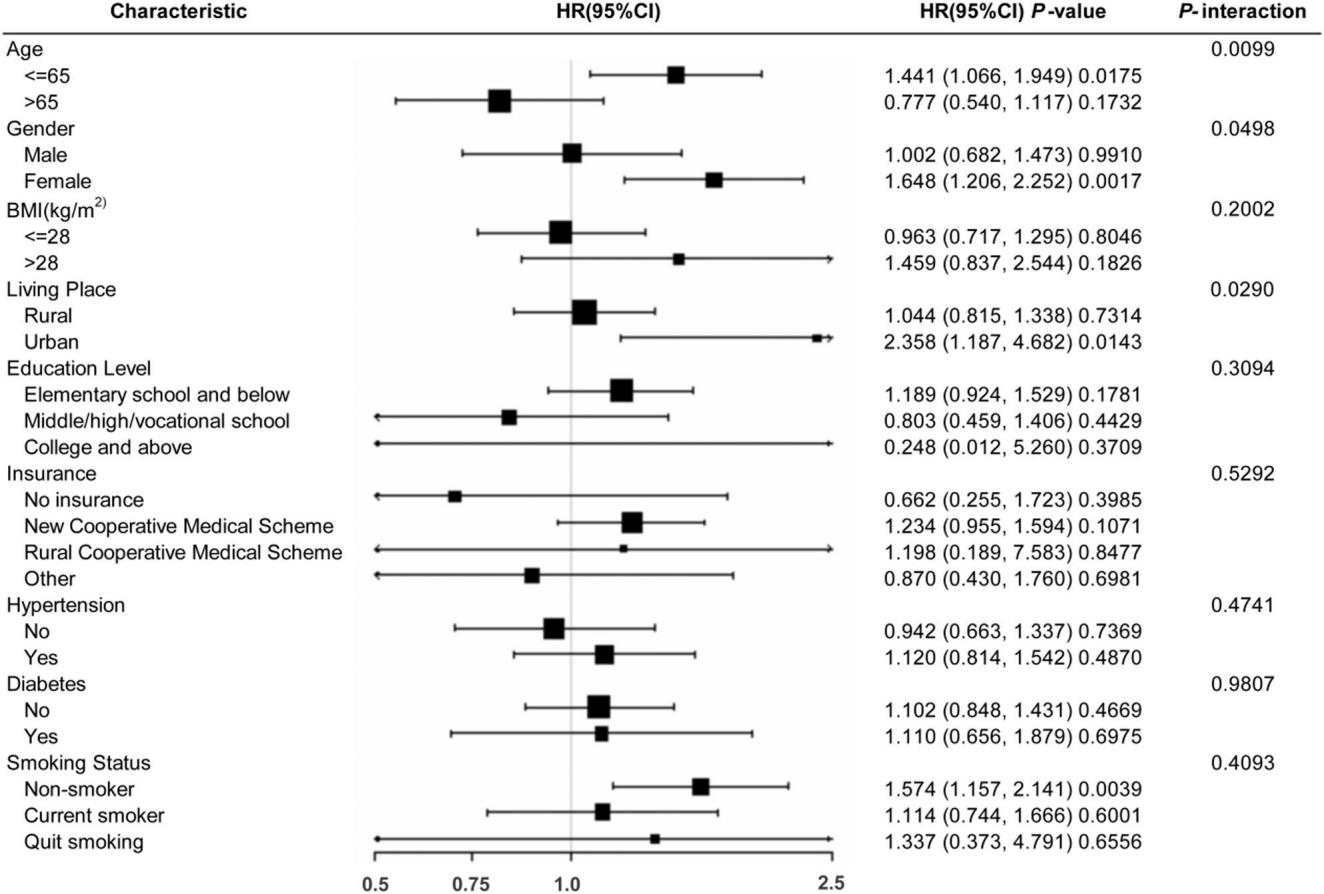
Subgroup analysis of the association between BRI and asthma risk. Stratified analyses were performed based on age, gender, BMI, living place, education level, insurance, hypertension, diabetes, and smoking status. The forest plot displays the Hazard Ratios (HR) and 95% Confidence Intervals (CI). P-interaction values indicate the statistical significance of the difference between subgroups

To further explore the stability of the association between BRI and asthma risk in different populations, we performed stratified analyses and interaction tests (Fig. 3). Significant interactions were observed for age (*P*-interaction = 0.0099), gender (*P*-interaction = 0.0498), and living place (*P*-interaction = 0.029). Specifically, the positive association between BRI and asthma was significant in participants aged ≤ 65 years (HR = 1.441, 95%CI: 1.066–1.949) but was attenuated and non-significant in those aged > 65 years. The risk was significantly elevated in females (HR = 1.648, 95% CI: 1.206–2.252), whereas no significant association was observed in males (HR = 1.002, 95% CI: 0.682–1.473). Participants living in urban areas showed a stronger association (HR = 2.358, 95% CI: 1.187–4.682) compared to rural residents. No significant interactions were detected for BMI, education level, insurance type, hypertension, diabetes, or smoking status (*P*-interaction > 0.05).

## Discussion

In this retrospective cohort study based on the nationally representative CHARLS database, we identified a significant non-linear association between the Body Roundness Index (BRI) and the risk of asthma among middle-aged and elderly Chinese adults. Our analysis of 7,754 participants revealed a U-shaped relationship characterized by a distinct threshold effect. Specifically, when BRI exceeded the inflection point of 4.12, the risk of asthma increased substantially (HR: 1.75, 95% CI: 1.34–2.27). Below this threshold, a lower BRI was associated with a reduced asthma risk (HR: 0.71, 95% CI: 0.54–0.92), reflecting the descending limb of the curve rather than a biologically independent protective effect. Notably, while the unadjusted analysis showed only a borderline association (*P* = 0.053), the fully adjusted models revealed a robust and highly significant relationship (*P* < 0.0001). This contrast underscores the critical role of confounding factors-such as age, gender, and lifestyle variables-in masking the true impact of central adiposity in crude analyses, thereby validating the necessity of multivariable adjustment to isolate the independent effect of BRI on respiratory health.

Obesity has established itself as a major global health concern and a significant risk factor for asthma [16, 26]. While Body Mass Index (BMI) is the traditional metric for general obesity [27], it has notable limitations in differentiating between muscle mass and adipose tissue, particularly in older adults where body composition shifts significantly [28, 29]. In contrast, BRI serves as a superior geometric index for estimating visceral adiposity, which is metabolically more active and pathogenic than subcutaneous fat [8, 30]. Our findings align with recent studies suggesting that body fat distribution, rather than total mass, is a more accurate predictor of respiratory outcomes. For instance, Wang et al. demonstrated a unidirectional causal relationship between body fat distribution and asthma risk [31], and Xu et al. found a positive association between the Visceral Adiposity Index (VAI) and asthma incidence in the US NHANES population [32]. Furthermore, research indicates that elevated BRI is strongly linked to accelerated lung function decline [21], likely due to the mechanical restriction of the diaphragm and the systemic pro-inflammatory state induced by visceral fat [33]. By utilizing a smoothed curve fitting analysis, our study extends these findings to the Chinese population, confirming that BRI captures the nuances of obesity-related asthma risk more effectively than traditional metrics.

A key strength of our analysis is the exploration of demographic heterogeneity. We observed a significant interaction with gender (*P*-interaction = 0.0498), where the association between elevated BRI and asthma risk was pronounced in females (HR = 1.648) but not significant in males. This gender-specific vulnerability aligns with the “female-predominant obesity asthma phenotype” often described in literature. The mechanism likely involves postmenopausal hormonal changes; the decline in estrogen accelerates visceral fat accumulation and alters adipose tissue metabolism [34]. This accumulation promotes the secretion of pro-inflammatory leptins and cytokines (e.g., IL-6), which exacerbate airway inflammation and hyperresponsiveness. Additionally, we found that the association was significant in the middle-aged and young-elderly group (≤ 65 years) but attenuated in the oldest-old (> 65 years). This suggests that the impact of body roundness on respiratory health is most critical in mid-life, whereas in advanced age, the “obesity paradox” or competing risks from other severe comorbidities may obscure this relationship.

We also conducted rigorous sensitivity analyses to ensure the robustness of our results. Given the complex interplay between obesity, respiratory health, and mental well-being, we adjusted for depressive symptoms using the CES-D 10 scale. The BRI-asthma association remained materially unchanged (HR = 1.126 for trend), indicating that mental health status did not confound the observed relationship in this cohort. This reinforces the conclusion that the link between body roundness and asthma is primarily driven by physiological and metabolic mechanisms rather than psychological confounders.

This study possesses several strengths, including the use of a large, nationally representative sample, the rigorous exclusion of baseline chronic pulmonary diseases to minimize reverse causality, and the application of advanced non-linear modeling. However, several limitations warrant acknowledgment. First, BRI was assessed based on a single measurement at baseline (2011). Although repeated measurements would ideally capture longitudinal changes in adiposity, baseline anthropometrics are widely accepted as valid proxies for long-term exposure in large epidemiological cohorts [35, 36], a method consistently employed in recent major studies [37]. Second, asthma diagnosis relied on self-reported physician diagnosis combined with ICD-coded medical records rather than objective spirometry. While this is a standard approach in large-scale surveys where lung function testing is not feasible, and has shown reasonable validity in CHARLS [24], some degree of misclassification remains possible. To mitigate this, we strictly excluded participants with COPD or chronic bronchitis. Any residual non-differential misclassification would likely bias the results toward the null, suggesting that our estimates may be conservative. Third, as an observational study, we can infer association but not causality. Finally, the study population was exclusively Chinese, so caution should be exercised when generalizing these findings to other ethnic groups with different body composition profiles.

In conclusion, this study demonstrates a significant, non-linear association between BRI and asthma risk among middle-aged and elderly adults in China, with a distinct risk threshold at a BRI of 4.12. The association is particularly strong in females and urban residents. These findings suggest that BRI is a valuable, easily obtainable anthropometric tool for identifying high-risk individuals in clinical and public health settings. Future strategies for asthma prevention in aging populations should consider targeting central obesity management, especially among postmenopausal women.

## Data Availability

The CHARLS datasets used in this study are available on request from their home page at http://charls.pku.edu.cn/. To download the data, approval from the CHARLS team is required.

## Abbreviations

BRI: Body Roundness Index
BMI: Body Mass Index
CHARLS: China Health and Retirement Longitudinal Study
ICD: International Classification of Diseases
WC: Waist Circumference
HR: Hazard Ratio
IC: Confidence Interval
VAI: Visceral Adiposity Index
NHANES: National Health and Nutrition Examination Survey
